# The association between passive smoking and sleep quality in a Chinese hypertensive population: A cross-sectional study

**DOI:** 10.18332/tid/176929

**Published:** 2024-02-02

**Authors:** Niuniu Sun, Yang Ni, Yuqian Deng, Jiale Qi, Zhenjie Yu, Chang Wu, Juan He, Yibo Wu

**Affiliations:** 1 School of Nursing, Henan University of Science and Technology, Luoyang, China; 2 Xiangya School of Nursing, Central South University, Changsha, China; 3 School of Journalism and Communication, Zhengzhou University, Zhengzhou, China; 4 School of Nursing, Tianjin Medical University, Tianjin, China; 5 School of Public Health, Peking University, Beijing, China

**Keywords:** hypertension, sleep quality, secondhand smoke, thirdhand smoke, tobacco smoke exposure

## Abstract

**INTRODUCTION:**

This study evaluates the association between passive smoking, specifically secondhand smoke (SHS) and thirdhand smoke (THS) exposure, and sleep quality in a hypertensive population.

**METHODS:**

We enrolled 1427 eligible hypertensive patients from a 2022 national cross-sectional survey in China. Data on tobacco smoke exposure and sleep were collected via questionnaires. Multiple logistic regression and linear regression were employed to assess the relationship between passive smoking and sleep quality characteristics, as well as the correlation between passive smoking exposure characteristics and sleep quality.

**RESULTS:**

Among 589 hypertensive patients with no tobacco smoke exposure, 679 exposed to SHS, and 159 exposed to THS, SHS exposure was associated with a higher risk of poor sleep quality, even after adjusting for potential confounding factors (β=0.10; 95% CI: 0.32–0.95). No significant relationship was observed between THS exposure and sleep quality. SHS exposure was associated with various sleep quality characteristics, including shorter sleep duration (AOR=1.71; 95% CI: 1.06–2.76) and increased frequency of 1–2 sleep disturbances per week (AOR=1.68; 95% CI: 1.25–2.26). Individuals exposed to SHS were more likely to experience poorer subjective sleep quality (AOR=1.53; 95% CI: 1.07–2.21) and have sleep efficiency <65% (AOR=1.82; 95% CI: 1.22–2.71). Exposure to passive smoking at home, in the community, in public places, exposure to passive smoking with family and friends, and increased frequency of exposure, were all associated with a higher risk of poor sleep quality.

**CONCLUSIONS:**

Our study suggests that SHS exposure in hypertensive populations is associated with poor sleep quality and various characteristics of sleep quality. No significant association was found between THS exposure and sleep quality. These findings underscore the need to enhance tobacco control efforts in China, particularly for individuals with chronic diseases, to safeguard public health.

## INTRODUCTION

Hypertension is one of the most common comorbidities globally and is a leading cause of premature death and cardiovascular disease^1^. The prevalence of hypertension in China is 23.2% (about 244.5 million people), with an increasing trend year after year, and another 41.3% (about 435.3 million people) are in occult hypertension^2^. China’s hypertensive population has seen an increase in awareness, treatment and control rates compared to previous years, but the overall rate is at a low level^3^. Unhealthy lifestyles contribute significantly to the growth of the population at risk for hypertension. Research has reported that sleep problems in pre-existing hypertension may trigger persistent cardiovascular neurologic dysregulation, which subsequently increases the risk of cardiovascular mortality^4^.

Tobacco is used by 22.3% of the world’s population^5^. There are 306 million tobacco smokers in China, with one-third of the world’s tobacco consumption^6^. The number of people who are passive smokers has exceeded 738 million^7^, and it is considered to be the 13th leading risk factor for death in China^8^. Passive smoking refers to secondhand smoke (SHS) and thirdhand smoke (THS). SHS is a major indoor pollutant, by which non-smokers are exposed to tobacco smoke from the smoking of others^9^. THS has recently been recognized as a new threat, and is the residual pollutants in the environment after the cigarette is extinguished, which adhere to the surface of objects, hair, and the surrounding environment^10^. It is widely recognized that tobacco smoke coexists with the human environment and has a broader impact by contributing to the burden of disease, especially ischemic heart disease^11^. A series of studies have also explored the potential risk factors of sleep quality issues caused by SHS^12-14^, where passive smoking triggers sleep deprivation and sleep disorders. To date, the negative effects of hypertension or SHS exposure on sleep have been well established. However, no research has found a correlation between passive smoking (including SHS and THS) and sleep quality in hypertensive patients, as sleep stability is a protective factor for reducing the prevalence of hypertension^15^. In light of this, the current collection of data on sleep quality in hypertensive populations was designed to investigate the association between passive smoking and sleep quality, as well as the relationship between passive smoking exposure characteristics and sleep quality, to provide a basis for effective treatment and prevention of blood pressure.

## METHODS

### Sampling and participants

The data are from a nationwide survey initiated by the School of Public Health at Peking University from 20 June to 31 August 2022, covering 148 cities, 202 districts and counties (excluding Hong Kong, Macao and Taiwan), 390 townships/towns/streets, and 780 neighborhoods/villages in 23 provinces, five autonomous regions, and four municipalities in mainland China. These sampling ratios were determined based on the ratio of the population provided by the Seventh National Population Census. A total sample size of 20000 people was estimated, with at least 500 to 2500 people sampled from each province. Participants were selected based on quota attributes such as gender and age at various levels of geographical locations.

Questionnaire sites were set up at health service centers or related health service stations in the sampled communities to conduct the survey. Participants were recruited through posters, paper or electronic invitations. The identity of all participants was verified, informed consent was obtained, and eligibility criteria were checked. The target participants were individuals aged >12 years who could understand the questionnaires and complete them independently or with the help of the investigators. Individuals with psychiatric disorders, cognitive impairment, those participating in similar studies, or those who did not wish to participate were excluded from the study. A total of 21916 questionnaires were collected^16^.

**Figure 1:**
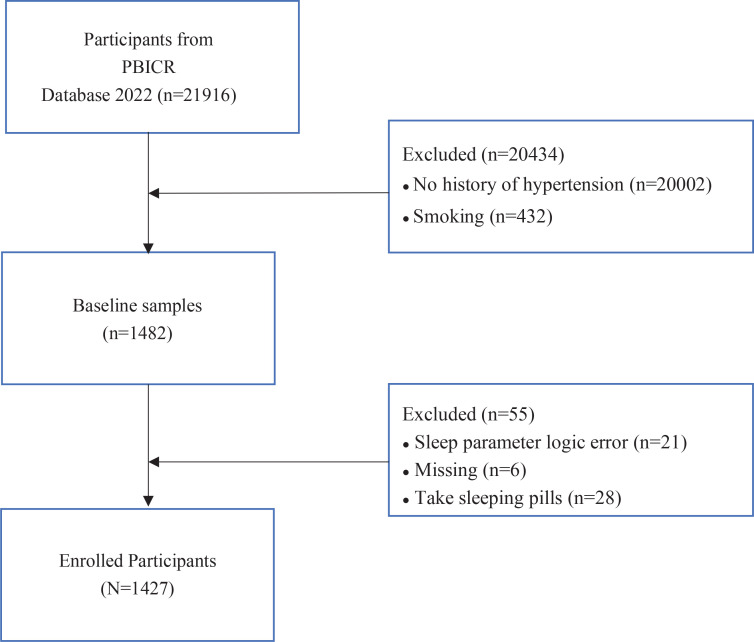
Participant selection process for this cross-sectional study of hypertensive individuals from a 2022 national cross-sectional survey in China

To calculate the required sample size, *a priori* sample size calculations were conducted for a one-way analysis of variance (ANOVA) using G*Power software version 3.1.9.6, considering an effect size of 0.25 and a statistical power of 0.95 at p<0.05^17^. A minimum of 252 samples was required. In our study, data from the participants without hypertension (n=20002) and who currently smoked cigarettes (n=432) were excluded; data from the participants who were on sleeping pills (n=28), who had cognitive difficulties (n=21) with the questionnaires, and with missing values (n=6), were further excluded to eliminate any possible effects on the sleep variable. Eventually, a sample size of n=1427 was chosen for the study to ensure sufficient statistical power (Figure 1). The study was reviewed and approved by the Ethics Committee of the Health Culture Research Center of Shaanxi, with project number JKWH-2022-02.

### Measurements of exposure variable

*Sleep quality*

Sleep quality was measured by a short version of the Pittsburgh Sleep Quality Index (B-PSQI) scale^17^. It consists of five components: sleep efficiency, sleep duration, sleep disturbance, sleep latency, and subjective sleep quality. The sleep quality score was defined as a continuous variable ranging 0–15, with higher scores associated with poorer sleep quality. The B-PSQI scale had a favorable sensitivity (75.82%) and specificity (76.99%) for categorizing poor sleep quality^18^. Thus, to further confirm whether there was a distributional difference in the quality of sleep between the different smoke-exposed groups, the total sleep quality scores were categorized into a dichotomous variable: 0–4 was categorized as good sleep quality, and ≥5 was categorized as poor sleep quality. Moreover, the brief form had adequate internal consistency (α=0.79). The Cronbach’s α for the B-PSQI in this study was 0.77.

### Smoke exposure and characteristics

The main independent variable was self-reported smoke exposure. The respondents were asked several questions: 1) ‘Do you have a smoking habit?’, with responses of ‘No’, ‘Used to’ and ‘Not now’; and 2) ‘Who are the people around you who have the habit of smoking?’, with multiple responses allowed being family members, co-workers, friends, and neighbors; and 3) ‘Do any of the above smokers smoke in your presence?’, with responses of ‘no one smokes’ for no exposure, ‘would’ for SHS exposure, and ‘would not’ for THS exposure.

The participants were asked about the area of exposure and duration of stay in the past seven days, with three levels according to the duration of smoke exposure: 1) 0 days, 2) 1–4 days/week, and 3) 5–7 days/week. The place of exposure was categorized according to the area of activities such as at home, in the work environment, in communities, and in public places.

### Sociodemographic information

We assessed general characteristics (age, gender, education level, employment status, marital status, body mass index (BMI, kg/m^2^), hypertension classification, hypertension complications, health behaviors (alcohol consumption, consumption of tea, taking antihypertensive drugs), and psychological factors (anxiety, depression). According to the Chinese Guidelines for the Management of Hypertension^19^, the systolic/diastolic blood pressure risk grading is categorized into three grades: Grade 1 = 140–159/90–99 mmHg, Grade 2 = 160–179/100–109 mmHg, and Grade 3 ≥180/100 mmHg. For the hypertension complications question (yes, no), participants were asked if they had coronary heart disease, chronic kidney disease, and stroke. Taking antihypertensive drugs and alcohol consumption versus consumption of tea were dichotomous variables (yes, no).

### Psychology variables

According to previous studies^20^, hypertensive individuals with depression and anxiety have a 2.03 and 1.89 times higher risk of poor sleep quality compared to hypertensive individuals without depression and anxiety, respectively. To control for the potential influence of psychological factors on sleep quality, the Generalized Anxiety Disorder-7 (GAD-7)^21^ was used to understand the participants’ psychological status by asking them about their experiences in the past two weeks. There are seven entries to assess anxiety symptoms with a total GAD score of 0–21. The Depression Screening Scale (Patient Health Questionaire-9 items, PHQ-9)^22^ is one of the international common depression detection scales. The PHQ-9 is a 9-item scale with a total score of 0–27. All of the above scales were scored using a 4-point Likert scale ranging 0–3 points; higher total scores indicate higher levels of depression and anxiety symptoms.

### Statistical analysis

Population characteristics, health behaviors, and psychological factors were delineated based on tobacco smoke exposure status using frequencies (n) and percentages (%), or median and interquartile range (IQR). Kruskal-Wallis tests were employed for non-normally distributed continuous variables and ordered data, while Pearson χ^2^ tests were applied for unordered data variables. The dependent variable, sleep quality, demonstrated approximate normal distribution, and differences in the impact of various tobacco smoke exposures were assessed using analysis of variance (ANOVA). Pairwise comparisons were conducted using the Bonferroni correction method. *A priori* and *post hoc* analyses were performed using G*Power software version 3.1.9.6 to evaluate the study design’s statistical power.

Differences in the distribution of tobacco smoke exposure and sleep quality characteristics, among the three groups, were compared using the rank sum test. Sleep characteristics with significant differences were investigated using univariate and multivariate logistic regression to study the relationship between passive smoking and sleep parameters. Factors statistically significant or related to sleep quality in the univariate analysis were selected as control variables for the multivariate analysis. In this way, three models were developed with progressively increasing controls for covariates, including Crude model with no adjustments; Model 1 was adjusted for gender, age, BMI, career status, education level, marital status, alcohol consumption, consumption of tea, and taking antihypertensive drugs; and Model 2 as for Model 1 plus anxiety and depression. In stepwise control of covariates, odds ratios (ORs) and 95% confidence intervals (CIs) were calculated. Due to the small number of ‘very poor subjective sleep quality’ in this study, we combined ‘very poor’ with ‘poor’ subjective sleep quality in subsequent analyses to ensure the robustness of the results.

Sensitivity analyses were conducted to test the robustness. The main relationship between passive smoking characteristics and sleep quality was re-examined using linear regression models (with SHS and THS as categorical variables) for trend testing of sleep quality as a continuous variable. After controlling for variables, the models yielded standardized coefficients (β) with 95% CI. A two-tailed p<0.05 was set as the significance level for all hypothesis tests, and SPSS 27.0 was used for data analysis.

## RESULTS

### Demographic characteristics

Conducting a *post hoc* statistical power analysis using G*Power for one-way ANOVA, we set the significance level (α) at 0.05 and effect size (d) at 0.12^17^. The computed power was found to be 0.98. This suggests statistical validity in our study. As shown in Table 1, 1427 non-smoking hypertensive participants were categorized into three exposure groups: 589 No Tobacco Smoke-Exposed (No TSE), 679 SHS, and 159 THS, individuals. Among them, females dominated the No TSE and THS groups (56.0%, 56.6%), while males constituted 50.7% in the SHS group. Median age across the groups was 63 years (IQR: 52–72). The SHS and THS exposure groups primarily comprised employed or retired individuals with higher educational levels and larger average BMI, compared to the No TSE group. Tea and alcohol consumption were more prevalent in SHS and THS groups. Exposure frequencies ranged from 1–4 days/week (46.8%, 40.3%) and 5–7 days/week (40.2%, 25.8%) for SHS and THS, respectively. Home-based SHS exposure was 40.6%, while community-based THS exposure was 33.5%. SHS primarily originated from family and friends (44.6%, 31.4%), whereas THS was mainly from family (53.9%). Notably, anxiety and depression levels were higher in SHS- and THS-exposed group than in the No TSE group, with anxiety scores of 4 (IQR: 0–7) and 4 (IQR: 1–7), and depression scores of 6 (IQR: 2–9) and 6 (IQR: 3–9), respectively.

**Table 1:** Sociodemographic characteristics of hypertensive patients from a 2022 national cross-sectional survey in China (N=1427)

*Characteristics*	*Total (N=1427) n (%)*	*No TSE (N=589) n (%)*	*SHS (N=679) n (%)*	*THS (N=159) n (%)*	*p^a^*
**Gender**					**0.036**
Male	672 (47.1)	259 (44.0)	344 (50.7)	69 (44.4)	
Female	755 (52.9)	330 (56.0)	335 (49.3)	90 (56.6)	
**Age** (years), median (IQR)	63 (52–72)	66 (58–73)	60 (49–70)	64 (49–74)	**<0.001**
**BMI** (kg/m^2^), median (IQR)	23.4 (21.5–25.9)	23.0 (21.2–25.4)	23.6 (21.8–26.0)	23.9 (21.8–26.0)	**0.004**
**Employment status**					**<0.001**
Employed	315 (22.1)	84 (14.3)	189 (27.8)	42 (26.4)	
Freelancing	192 (13.5)	71 (12.1)	107 (15.8)	14 (8.8)	
Unoccupied	379 (26.5)	172 (29.2)	167 (24.6)	40 (25.2)	
Retired	541 (37.9)	262 (44.5)	216 (31.8)	63 (39.6)	
**Education level**					**<0.001**
Primary school and lower	542 (38.0)	253 (43)	238 (35.1)	51 (32.1)	
Middle school	291 (20.4)	125 (21.2)	138 (20.3)	28 (17.6)	
High school	168 (11.8)	66 (11.2)	87 (12.8)	15 (9.4)	
University and higher	426 (29.8)	145 (24.6)	216 (31.8)	65 (40.9)	
**Marital status**					**<0.001**
Unmarried	41 (2.9)	7 (1.2)	26 (3.8)	8 (5.0)	
Married	1186 (83.1)	480 (81.5)	580 (85.4)	126 (79.2)	
Divorced/widowed	200 (14.0)	102 (17.3)	73 (10.8)	25 (15.7)	
**Alcohol consumption**					**<0.001**
No	1204 (84.4)	537 (91.2)	533 (78.5)	134 (84.3)	
Yes	223 (15.6)	52 (8.8)	146 (21.5)	25 (15.7)	
**Consumption of tea**					**0.002**
No	732 (51.3)	334 (56.7)	328 (48.3)	70 (44.0)	
Yes	695 (48.7)	255 (43.3)	351 (51.7)	89 (56.0)	
**Taking antihypertensive drugs**					0.234
No	596 (41.8)	260 (44.1)	268 (39.5)	68 (42.8)	
Yes	831 (58.2)	329 (55.9)	411 (60.5)	91 (57.2)	
**Hypertension classification**					0.53
Grade 1	953 (66.8)	379 (64.3)	466 (68.6)	108 (67.9)	
Grade 2	414 (29.0)	181 (30.8)	188 (27.7)	45 (28.3)	
Grade 3	60 (4.2)	29 (4.9)	25 (3.7)	6 (3.8)	
**Complications of hypertension**					0.12
No	1084 (76.0)	443 (75.2)	529 (77.9)	112 (70.4)	
Yes	343 (24.0)	146 (24.8)	150 (22.1)	47 (29.6)	
**Exposure time** (days/week)					**<0.001**
0	142 (16.9)		88 (13)	54 (34)	
1–4	382 (45.6)		318 (46.8)	64 (40.3)	
5–7	314 (37.5)		273 (40.2)	41 (25.8)	
**Exposure by social environment^b^**					**<0.001**
Family	510 (46.2)		413 (44.6)	97 (53.9)	
Neighbors	262 (23.7)		222 (24)	40 (22.2)	
Friends	333 (30.1)		290 (31.4)	43 (23.9)	
**Exposure sites^b^**					**<0.001**
Home	477 (39.1)		415 (40.6)	62 (31)	
Workplace	292 (23.9)		250 (24.5)	42 (21)	
Community	304 (24.9)		237 (23.2)	67 (33.5)	
Public areas	148 (12.1)		119 (11.7)	29 (14.5)	
**Psychological factors**					
Anxiety, median (IQR)	4 (0–7)	3 (0–7)	4 (0–7)	4 (1–7)	**0.019**
Depression, median (IQR)	5 (2–9)	5 (1–9)	6 (2–9)	6 (3–9)	**<0.001**

IQR: interquartile range. No TSE: no tobacco smoke exposure. SHS: secondhand smoke. THS: thirdhand smoke.

a Differences in percentage or mean differences analyzed between groups of the three tobacco smoke exposures by Pearson χ^2^ tests and the Kruskal-Wallis test, respectively.

b Multiple responses permitted. Statistically significant differences between groups of the three tobacco smoke exposures, at p<0.05.

### Differences in sleep parameters

**Figure 2:**
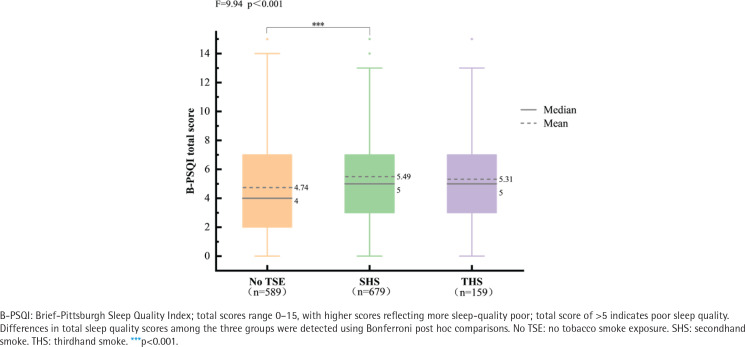
Differences in total sleep quality scores between the three smoke exposure groups were compared by ANOVA, hypertensive individuals from a 2022 national cross-sectional survey in China (N=1427)

The ANOVA results in Figure 2 support our hypothesis, indicating an association between smoke exposure and sleep quality across all three groups (F=9.94, p<0.001). Utilizing the Bonferroni correction method for pairwise comparisons, we found that poor sleep quality was significantly higher in the SHS-exposed group compared to the No TSE group (p<0.001), with no significant differences in sleep quality observed between the THS-exposed group and the no TSE group, as well as between the SHS exposed group. To explore gender differences in sleep quality among hypertensive individuals exposed to tobacco smoke, the data in Supplementary file Table A1 suggest that, in the gender-based analysis, in comparison to good sleep quality, there was no observable gender difference in poor sleep quality.

Table 2 shows the differences in comparing the three groups of smoke exposure with the characteristics of sleep quality. Regarding the total sleep quality score (B-PSQI >5), the ratio of poor passive smoking sleep quality was significantly higher in both groups than in the No TSE group. The sleep duration in the SHS-exposed and THS-exposed groups was <7 hours; 26.1% of the SHS-exposed and 19.5% of the THS-exposed group had 1–2 sleep disturbances per week. Compared with very good subjective sleep, the vast majority of SHS-exposure and THS-exposure individuals had good subjective sleep quality. In contrast, 21.1% of SHS-exposure individuals had poor subjective sleep quality. And SHS and THS sleep efficiency was insufficient, with 13.0% SHS-exposed and 13.8% THS-exposed sleep efficiency <65%.

**Table 2:** Differences in sleep parameters between different smoke exposures in hypertensive individuals from a 2022 national cross-sectional survey in China (N=1427)

*Variables*	*Total (N=1427) n (%)*	*No TSE (N=589) n (%)*	*SHS (N=679) n (%)*	*THS (N=159) n (%)*	*p*
**Sleep quality total score**					**0.037**
Good (0–4)	674 (47.2)	302 (51.3)	301 (44.3)	71 (44.7)	
Poor (≥5)	753 (52.8)	287 (48.7)	378 (55.7)	88 (55.3)	
**Sleep duration** (h)					**<0.001**
>7	448 (31.4)	229 (38.9)	171 (25.2)	48 (30.2)	
6–7	550 (38.5)	211 (35.8)	282 (41.5)	57 (35.8)	
5–6	339 (23.8)	116 (19.7)	180 (26.5)	43 (27.0)	
<5	90 (6.3)	33 (5.6)	46 (6.8)	11 (6.9)	
**Sleep disturbances** (per week)					**0.028**
None in the past month	553 (38.8)	244 (41.4)	247 (36.4)	62 (39.0)	
<1	405 (28.4)	177 (30.1)	180 (26.5)	48 (30.2)	
1 or 2	320 (22.4)	112 (19.0)	177 (26.1)	31 (19.5)	
≥3	149 (10.4)	56 (9.5)	75 (11.0)	18 (11.3)	
**Subjective sleep quality**					**0.045**
Very good	294 (20.6)	141 (23.9)	120 (17.7)	33 (20.8)	
Good	849 (59.5)	335 (56.9)	416 (61.3)	98 (61.6)	
Poor	284 (19.9)	113 (19.2)	143 (21.1)	28 (17.6)	
**Sleep latency** (min)					0.097
≤15	525 (36.8)	234 (39.7)	235 (34.6)	56 (35.2)	
16–30	593 (41.5)	240 (40.7)	288 (42.4)	65 (40.9)	
31–60	215 (15.1)	81 (13.8)	106 (15.6)	28 (17.6)	
>60	94 (6.6)	34 (5.8)	50 (7.4)	10 (6.3)	
**Sleep efficiency** (%)					**0.004**
>85	402 (28.1)	186 (31.6)	175 (25.8)	41 (25.8)	
75–84	591 (41.4)	249 (42.3)	281 (41.4)	61 (38.4)	
65–74	272 (19.1)	102 (17.3)	135 (19.9)	35 (22.0)	
<65	162 (11.4)	52 (8.8)	88 (13.0)	22 (13.8)	

No TSE: no tobacco smoke exposure. SHS: secondhand smoke. THS: thirdhand smoke. Difference in percentage differences analyzed between the three tobacco smoke exposures by the Kruskal-Wallis test. Statistical significance, at p<0.05.

### The association between smoke exposure status and sleep quality

Using unadjusted single-factor logistic regression analyses and adjusted multifactorial logistic regression analyses, the associations with sleep dimensions in hypertensive populations stratified by passive smoking are presented in Table 3. In unadjusted analyses, SHS-exposed individuals were likely to experience short sleep duration (OR=1.71; 95% CI: 1.32–2.20), low sleep efficiency (OR=1.54; 95% CI: 1.07–2.23), and 1–2 sleep disturbances per week (OR=1.53; 95% CI: 1.16–2.02). Compared with very good subjective sleep quality, SHS-exposed individuals had good subjective sleep quality (OR=1.39; 95% CI: 1.07–1.82) and poor subjective sleep quality (OR=1.47; 95% CI: 1.06–2.04). In Model 2, SHS exposure was associated with sleep duration of 6–7 h (AOR=1.54; 95% CI: 1.18–2.00), 5–6 h (AOR=1.64; 95% CI: 1.21–2.21), and <5 h (AOR=1.71; 95% CI: 1.06–2.76). SHS exposure resulted in 1–2 sleep disturbance per week (AOR=1.68; 95% CI: 1.25–2.26), reports of good sleep quality (AOR=1.44; 95% CI: 1.08–1.92), reports of poor sleep quality (AOR=1.53; 95% CI: 1.07–2.21), and sleep efficiency <65% (AOR=1.82; 95% CI: 1.22–2.71). Other than that, this study did not find any association between THS exposure and various sleep indicators.

**Table 3:** Multiple logistic regression analysis of the relationship between passive smoking and sleep parameters in hypertensive individuals from a 2022 national cross-sectional survey in China (N=1427)

*Characteristics*	*Secondhand smoke exposure*						*Thirdhand smoke exposure*					
	*Crude model*		*Model 1*		*Model 2*		*Crude model*		*Model 1*		*Model 2*	
	*OR (95% CI)*	*p*	*AOR (95% CI)*	*p*	*AOR (95% CI)*	*p*	*OR (95% CI)*	*p*	*AOR (95% CI)*	*p*	*AOR (95% CI)*	*p*
**Sleep duration** (h)												
>7 ®	1		1		1		1		1		1	
6–7	1.71 (1.32–2.20)	<0.001	1.52 (1.17–1.98)	0.002	1.54 (1.18–2.00)	**0.002**	0.96 (0.64–1.45)	0.86	0.91 (0.60–1.38)	0.65	0.92 (0.61–1.40)	0.69
5–6	1.83 (1.38–2.44)	<0.001	1.68 (1.25–2.26)	<0.001	1.64 (1.21–2.21)	**0.001**	1.21 (0.78–1.88)	0.39	1.13 (0.72–1.77)	0.59	1.09 (0.69–1.71)	0.72
<5	1.69 (1.07–2.67)	<0.001	1.80 (1.12–2.89)	0.015	1.71 (1.06–2.76)	**0.029**	1.16 (0.58–2.33)	0.68	1.03 (0.50–2.09)	0.95	0.97 (0.47–2.01)	0.94
**Sleep disturbances** (per week)												
None in the past month ®	1		1		1		1		1		1	
<1	0.99 (0.77–1.28)	0.95	1.05 (0.80–1.37)	0.74	1.02 (0.77–1.34)	0.91	1.07 (0.71–1.59)	0.76	1.03 (0.68–1.54)	0.91	0.99 (0.65–1.49)	0.94
1 or 2	1.53 (1.16–2.02)	0.002	1.73 (1.29–2.31)	<0.001	1.68 (1.25–2.26)	**<0.001**	0.85 (0.54–1.34)	0.48	0.83 (0.52–1.32)	0.43	0.78 (0.49–1.25)	0.30
≥3	1.26 (0.87–1.80)	0.22	1.39 (0.95–2.03)	0.09	1.31 (0.88–1.96)	0.18	1.09 (0.62–1.90)	0.77	1.09 (0.62–1.94)	0.76	0.93 (0.51–1.71)	0.82
**Subjective sleep quality**												
Very good ®	1		1		1		1		1		1	
Good	1.39 (1.07–1.82)	0.016	1.51 (1.14–2.00)	0.004	1.44 (1.08–1.92)	**0.013**	1.03 (0.68–1.57)	0.88	1.07 (0.70–1.63)	0.77	0.98 (0.63–1.51)	0.93
Poor	1.47 (1.06–2.04)	0.022	1.65 (1.17–2.32)	0.005	1.53 (1.07–2.21)	0.021	0.87 (0.51–1.47)	0.59	0.87 (0.50–1.49)	0.6	0.72 (0.41–1.28)	0.26
**Sleep efficiency** (%)												
>85 ®	1		1		1		1		1		1	
75–84	1.18 (0.91–1.52)	0.21	1.23 (0.94–1.61)	0.13	1.22 (0.93–1.59)	0.16	1.01 (0.67–1.54)	0.95	0.98 (0.64–1.50)	0.93	0.96 (0.63–1.47)	0.85
65–74	1.28 (0.94–1.74)	0.12	1.37 (0.99–1.89)	0.06	1.34 (0.97–1.85)	0.08	1.30 (0.81–2.10)	0.28	1.26 (0.77–2.05)	0.36	1.21 (0.74–1.97)	0.46
<65	1.54 (1.07–2.23)	0.02	1.92 (1.30–2.83)	**0.001**	1.82 (1.22–2.71)	**0.003**	1.38 (0.80–2.41)	0.25	1.27 (0.72–2.24)	0.42	1.18 (0.66–2.11)	0.58

AOR: adjusted odds ratio. Crude model: no adjustments. Model 1: adjusted for gender, age, BMI, employment status, education level, marital status, alcohol consumption, consumption of tea, and take antihypertensive drugs. Model 2: adjusted as for Model 1 plus anxiety and depression. ® Reference categories. Statistical significance, at p<0.05.

Table 4 shows the unadjusted and adjusted associations between different smoke exposure characteristics and sleep quality analyzed using multiple linear regression. The No TSE group was negatively associated with sleep quality, suggesting that the No TSE group had good sleep quality. The SHS-exposed group was positively associated with poor sleep quality (β=0.10; 95% CI: 0.32–0.95), while the THS-exposed was not significantly associated with sleep quality. After adjusting for a range of confounders, the No TSE group remained stable in sleep quality. Meanwhile, Exposure to passive smoking, including contact with family (β=0.07; 95% CI: 0.12–0.76) and friends (β=0.06; 95% CI: 0.21–0.78), as well as exposure at home (β=0.06; 95% CI: 0.05–0.69), in the community (β=0.07; 95% CI: 0.12–0.87), and in public areas (β=0.05; 95% CI: 0.02–1.01), was associated with sleep quality. In addition, compared with passive smoking zero days per week, there was a slight increase in sleep quality in hypertensive patients exposed to smoke 1–4 days per week (β=0.07; 95% CI: 0.11–0.80), and 5–7 days per week (β=0.06; 95% CI: 0.09–0.74).

**Table 4:** Multiple linear regression analysis of the relationship between smoke characteristics and sleep quality of hypertensive individuals with smoke exposure status, in a 2022 national cross-sectional survey in China (N=1427)

*Variable*	*Crude model*		*Model 1*		*Model 2*	
	*β (95% CI)*	*p*	*β (95% CI)*	*p*	*β (95% CI)*	*p*
**Smoke exposure status**						
No TSE	-0.12 (-1.04 – -0.4)	<0.001	-0.13 (-1.16 – -0.50)	<0.001	-0.10 (-0.96 – -0.33)	**<0.001**
SHS	0.10 (0.32–0.95)	<0.001	0.12 (0.41–1.06)	<0.001	0.10 (0.32–0.93)	**<0.001**
THS	0.02 (-0.33–0.68)	0.51	0.02 (-0.35–0.66)	0.55	-0.002 (-0.50–0.46)	0.93
**Exposure by social environment**						
Family	0.11 (0.35–1.01)	<0.001	0.10 (0.27–0.95)	<0.001	0.07 (0.12–0.76)	**0.01**
Colleagues	0.001 (0.43–0.44)	0.98	0.03 (-0.21–0.76)	0.27	0.03 (-0.25–0.67)	0.37
Neighbors	0.03 (-0.18–0.65)	0.26	0.04 (-0.13–0.70)	0.18	0.03 (-0.12–0.71)	0.25
Friends	0.04 (-0.12–0.63)	0.19	0.07 (0.07–0.87)	0.02	0.06 (0.21–0.78)	**0.04**
**Exposure place**						
Home	0.08 (0.20–0.87)	0.002	0.07 (0.13–0.81)	0.007	0.06 (0.05–0.69)	**0.03**
Workplace	0.04 (-0.11–0.68)	0.16	0.07 (0.09–0.94)	0.018	0.04 (-0.08–0.72)	0.12
Community	0.06 (0.08–0.86)	0.018	0.08 (0.18–0.97)	0.004	0.07 (0.12–0.87)	**0.01**
Public areas	0.07 (0.15–1.19)	0.012	0.08 (0.25–1.30)	0.004	0.05 (0.02–1.01)	**0.04**
**Exposure time** (days/week)						
0	-0.11 (-1.00 – -0.38)	<0.001	-0.12 (-1.07 – -0.42)	<0.001	-0.10 (-0.93 – -0.32)	**<0.001**
1–4	0.07 (0.14–0.86)	0.006	0.09 (0.23–0.96)	0.001	0.07 (0.11–0.80)	**0.01**
5–7	0.06 (0.04–0.81)	0.03	0.05 (0.006–0.77)	0.046	0.06 (0.09–0.74)	**0.04**

No TSE: no tobacco smoke exposure. SHS: secondhand smoke. THS: thirdhand smoke. Crude model: no adjustments. Model 1: adjusted for gender, age, BMI, employment status, education level, marital status, alcohol consumption, consumption of tea, and take antihypertensive drugs. Model 2: adjusted as for Model 1 plus anxiety and depression. β: standardized coefficient. Statistical significance, at p<0.05.

## DISCUSSION

In our national survey we observed that 52.8% of hypertensive patients had poor sleep quality, higher than previously reported. The prevalence of poor sleep quality in hypertensive patients as measured by PSQI ranged from 35.5% to 52.5% in different studies^23,24^, and we conclude that our results are higher than previously reported. This suggests that the hypertensive population is prone to sleep problems, and we need to explore the risk factors affecting sleep. After further exclusion of confounders in the present study, SHS is considered one of the important risk factors affecting sleep quality in hypertensive individuals. To the best of our knowledge, this is the first study to report the relationship between passive smoking and sleep quality in a hypertensive population.

Sleep quality is a comprehensive sleep assessment indicator that includes sleep duration and other sleep manifestations. Although most studies focus on the sleep quality of healthy individuals^13,25,26^, there is still insufficient research on the relationship between SHS exposure and sleep quality in patients with chronic diseases. In this context, we observed that hypertensive males exposed to SHS had good sleep quality, which individual differences, lifestyle, and psychological factors may influence. However, there was no gender difference in poor sleep quality. This suggests that we may have similar effects of passive smoking on sleep quality across genders, but the exact mechanism still needs further research. Meanwhile, SHS exposure in hypertensive patients may be associated with multiple aspects of sleep quality. Specifically, for adults aged 18–64 years, the recommended sleep time is 7–9 hours; for elderly people aged ≥65 years, it should be 7–8 hours^27^. Research has found that compared to individuals without TSE, hypertensive patients exposed to SHS have significantly reduced sleep time, leading to a decrease in their sleep efficiency and subjectively experiencing a decrease in sleep quality. This conclusion is consistent with previous measurements based on objective biomarkers of tobacco smoke; an increase in urinary cotinine levels has been associated with abnormal sleep time, difficulty maintaining sleep, and various other sleep problems^28^. Furthermore, this impact on sleep may be related to nicotine in tobacco smoke. Nicotine has an excitatory effect, which can stimulate the synaptic transmission of acetylcholine and glutamate, thereby promoting the release of dopamine and causing certain effects on the central nervous system. Both long-term and acute exposure to tobacco smoke can interfere with the expression of clock genes in the human body, disrupt the inherent biological clock^29^, and thus affect sleep quality. Therefore, for the treatment and management of hypertensive patients, in addition to traditional medication and lifestyle interventions, more attention is needed to ascertain the causes of their sleep problems.

Current evidence on the relationship between exposure to passive smoking, primarily SHS, and sleep quality is inconsistent and controversial. However, many studies are consistent with our results, as in that sleep deprivation occurs about 1.77 times more in multiracial US adults exposed to SHS^12^. SHS exposure is negatively associated with sleep quality in never smoking middle-aged adults in Northwest China^30^. In a study using the PSQI scale, youth exposure to environmental tobacco smoke exposure was found to be positively associated with poor sleep quality^14^. However, some studies found the opposite result, with a British study of 498208 participants finding^31^ that excessive sleep duration (>9 h) is more likely to occur with passive smoking in the presence of high levels of smoke exposure and that passive smoking is associated with sleep disorders. The reason for the contrasting sleep duration results with our study may lie in the differences in sleep duration subgroups and the possible susceptibility of sleep to smoke disruption and recurrent refreshing of sleep at high levels of exposure. This study did not find an association between THS and sleep quality. The reason for this discrepancy with our original hypothesis may be that the THS exposure sample size was small. Based on the exposure characteristics of THS, the tobacco smoke particles retained by THS are more concealed than those of SHS. After being converted into toxic compounds, these particles will accumulate over time on surfaces that are easily adsorbed, such as carpets, floors, and sofas^10^. In addition, the primary route of exposure to THS chemicals is through hand-to-mouth transmission, making infants and school-age children the most sensitive to the adverse health effects of THS^9^. To further expand the research field and explore the impact of THS exposure on different populations, especially chronic disease groups represented by hypertension, we encourage longitudinal studies. This will help elucidate the potential complex correlation mechanism between THS and sleep quality over time in hypertensive patients, providing a scientific basis for prevention and treatment.

In addition, our investigation shows that sleep quality is associated with exposure places, social environment, and frequency. Firstly, exposure to passive smoking is mainly at home, in communities, and public places. Secondly, exposure groups are those close to non-smokers, including families and friends. Finally, increased frequency of exposure is significantly associated with sleep quality. Young people in the US report more sleep problems when exposed to medium and high levels of smoke than low levels of smoke^32^. Other results are consistent with previous prevalence surveys of passive smoking^33^. Passive smokers are susceptible to exposure to respirable hazardous substances in their homes, neighborhoods, and public places. For tobacco smoke that occurs in the home and is inhaled by an acquaintance, in addition to the close relationship between the two parties, sharing and giving cigarettes is considered a courtesy to others in China^34^, making it difficult for non-smokers to stop the smoker’s behavior. At the same time, passive smoking participants in both groups of this study tended to have alcohol consumption behaviors, which means smoking and alcohol consumption behaviors often coexist. Consequently, guests invited to the home for alcohol consumption may be accompanied by smoking behavior, which increases the exposure of non-smokers to SHS^33^. However, individuals themselves may lack awareness regarding the health risks associated with passive smoking. While some smokers choose not to smoke in communities to protect non-smokers from secondhand smoke exposure, they may overlook the presence of thirdhand smoke. Currently, most cities in China are still in a situation where the ban on smoking in public places is an advocacy rule, and its influence is relatively weak^35^. Creating tobacco control and smoke-free environments requires the support and care of individuals, families, and society in all aspects. Private spaces such as homes should be promoted as ‘smoke-free’. Smokers should be educated about tobacco, and smoke-free concepts should be incorporated into family education. Medical staff should take the initiative to inform the harms of smoking, and non-smokers need to know whether family members have smoking behaviors and educate both parties about tobacco control. Creating a sense of a smoke-free community in communities psychologically influences smokers’ view of health so that they consciously comply with the ban on smoking in public places.

### Limitations

There are limitations to this study. Firstly, the limited sample size and the exclusive focus on the Chinese population restrict the generalizability of the study results to other countries. Secondly, objective indicators did not validate self-reported smoke exposure and sleep information, and there were limitations to the accuracy and validity of the relevant information. However, resources, especially objective measurement tools, are scarce when it comes to generalized surveys, and self-report questionnaires are more commonly and economically used than other assessment^36^. This study can still provide some reference for the effective treatment and prevention of hypertension. The conclusions reached in this study are consistent with the most used PSQI results. Thirdly, the cross-sectional design could only establish associations, making it difficult to determine the causal relationship between passive smoking and sleep quality in hypertensive populations.

## CONCLUSIONS

Our study suggests that SHS exposure in hypertensive populations is associated with poor sleep quality and various dimensions of sleep quality. However, THS exposure is not associated with sleep quality. In addition, we found that the location of exposure, the social environment exposed to, and the frequency of exposure increased the likelihood of poor sleep quality. These findings suggest that as the prevalence of hypertension continues to rise, we need to further explore and understand the lifestyle and behavioral factors that affect hypertension. Research shows that tobacco control has a positive effect on maintaining the sleep quality of hypertensive patients, thereby helping to reduce the incidence of hypertension. Therefore, China should increase its efforts and speed in tobacco control to protect the health of its citizens, especially those with chronic diseases.

## Data Availability

The data supporting this research are available from the authors on reasonable request.
