# Supplementary material for: The association between passive smoking and sleep quality in a Chinese hypertensive population: A cross-sectional study
Source: Tob Induc Dis. 2024 Feb 2;22:10.18332/tid/176929. doi: 10.18332/tid/176929 (PMC10835764; doi:10.18332/tid/176929)
Supplement: Supplementary file 1 [file TID-22-30-s1.pdf]

SUPPLEMENTARY TABLE

Appendix Table A1: Comparison of Gender Differences in Sleep Quality among Smoke Exposure Groups in hypertensive individuals from a 2022 national cross-sectional survey in China (n=1 427)

**Categorical Variables**	**Total**	**No TSE**		**SHS**		**THS**		**P Value[a]**
		male	female	male	female	male	female	
Sleep quality total score（%）								
Good 0-4	674	133(39.3)	169(50.3)	173(51.2)	128(38.1)	32(9.5)	39(11.6)	**0.03**
Poor > 5	753	126(37.7)	161(38.4)	171(51.2)	207(49.4)	37(11.1)	51(12.2)	0.85

Note: %: the percentage of subjects， No TSE: no tobacco smoke exposure， SHS: second-hand smoke , THS: third-hand smoke
Significant difference between groups of three tobacco smoke exposure, statistical significance (p-value < 0.05)
a Difference in percentage or mean differences analyzed between the three tobacco smoke exposure by the Pearson x^2 tests

©2024 Sun N. et al.
